# High-Quality Draft Genome Sequence of *Burkholderia contaminans* CH-1, a Gram-Negative Bacterium That Metabolizes 2-Azahypoxanthine, a Plant Growth-Regulating Compound

**DOI:** 10.1128/genomeA.01148-17

**Published:** 2017-10-12

**Authors:** Jae-Hoon Choi, Hikaru Sugiura, Ryota Moriuchi, Hirokazu Kawagishi, Hideo Dohra

**Affiliations:** a Research Institute of Green Science and Technology, Shizuoka University, Shizuoka, Japan; b College of Agriculture, Academic Institute, Shizuoka University, Shizuoka, Japan; c Graduate School of Science and Technology, Shizuoka University, Shizuoka, Japan; d Department of Biological Science, Graduate School of Integrated Science and Technology, Shizuoka University, Shizuoka, Japan

## Abstract

*Burkholderia contaminans* strain CH-1 converts 2-azahypoxnathine to 2-aza-8-oxohypoxanthine, plant growth-regulating compounds, by oxidation. We report here the high-quality draft genome sequence of *B. contaminans* CH-1. The genome contains 8,065 protein-coding sequences, including several genes possibly involved in metabolizing 2-azahypoxanthine.

## GENOME ANNOUNCEMENT

*Burkholderia contaminans* belongs to the *Burkholderia cepacia* complex, which comprises approximately 20 closely related species (1). We recently reported that *B. contaminans* strain CH-1, isolated from an airborne-contaminated nutrient medium, showed a high level of activity in converting 2-azahypoxanthine (AHX) to 2-aza-8-oxohypoxanthine (AOH). Both compounds were novel plant growth-regulating compounds related to fairy ring formation (2).

Genomic DNA of *B. contaminans* strain CH-1 was extracted using the DNeasy blood and tissue kit and fragmented using the Covaris acoustic solubilizer. A paired-end library constructed by the TruSeq DNA PCR-free library preparation kit was sequenced using the Illumina MiSeq platform (301-bp paired-end) at the Instrumental Research Support Office, Research Institute of Green Science and Technology, Shizuoka University. The raw read sequences were cleaned up using Trimmomatic (3) by trimming adapter sequences, low-quality ends (quality score, <15), the last 301 bases, and reads less than 150 bp. The resultant 2,052,682 high-quality read pairs, totaling 1,012 Mb and corresponding to an approximately 114-fold coverage of the genome, were assembled using SPAdes version 3.10.0 (4) with a default set of *k*-mer sizes and options (–careful, –only-assembler, and –cov-cutoff auto); contigs less than 200 bp were eliminated. Average nucleotide identity (ANI) analysis (5, 6) of the resultant 94 contigs showed an extremely high ANI value (99.98%) with the improved high-quality draft genome of *B. contaminans* LMG 23361 (GenBank accession number MCAU02000000) (7). 

To construct scaffolds of the CH-1 genome, the contigs were aligned to the LMG 23361 genome sequence using CONTIGuator version 2.7 (8). The pseudocontigs were manually curated by alignments with contigs and the LMG 23361 genome, using BLAST and Geneious version 10.2.2 (9), and then circularized using Circlator version 1.1.2 (10). The high-quality draft genome of *B. contaminans* CH-1 contained three circular chromosomes, in which only five gaps remained, as well as a putative linear plasmid (designated pBC453), totaling 8,901,643 bp with a G+C content of 66.0%. The CH-1 genome was annotated using the DFAST-core stand-alone program version 0.9.4 (11) and manually curated. The CH-1 genome contains 8,065 protein-coding sequences, including 85 possible pseudogenes (1.05%), 18 rRNAs (6 clusters of 5S, 16S, and 23S rRNAs), and 82 tRNAs. The proteins encoded by the CH-1 genome were additionally annotated using BlastKOALA (12) to characterize protein functions and reconstruct the purine metabolism pathway in the KEGG database (13). Among 8,065 proteins, 82 were assigned to the purine metabolism pathway (ko00230), including the xanthine dehydrogenase small subunit (XdhA, K13481) and large subunit (XdhB, K13482), which are possibly involved in the conversion of AHX to AOH, as predicted by the purine metabolism pathway in plants (14). In addition, three proteins were assigned to the xanthine dehydrogenase accessory factor (XdhC, K07402), which is required for xanthine dehydrogenase activity in *Rhodobacter capsulatus* (15). These genome data will help elucidate the mechanism for converting AHX to AOH and improve methods for the large-scale production of AOH for its practical use in agriculture.

### Accession number(s).

The high-quality draft genome sequence of *B. contaminans* CH-1 has been deposited in DDBJ/ENA/GenBank under the accession numbers AP018357 to AP018360 (four entries).
